# The nasty neighbour in the striped mouse (*Rhabdomys pumilio*) steals paternity and elicits aggression

**DOI:** 10.1186/1742-9994-7-19

**Published:** 2010-06-23

**Authors:** Carsten Schradin, Carola Schneider, Anna K Lindholm

**Affiliations:** 1 Institute of Evolutionary Biology and Environmental Studies, Department of Animal Behaviour, University of Zurich, Winterthurerstr. 190, 8057 Zurich, Switzerland; 2 School of Animal, Plant and Environmental Sciences, University of the Witwatersrand, Johannesburg, South Africa; 3 University of Muenster, Department of Behavioural Biology, Badestr. 9, 48149 Münster, Germany

## Abstract

**Background:**

Territoriality functions to monopolize access to resources including mates, but is costly in terms of energy and time investment. Some species reduce these costs by being less aggressive towards their neighbours than towards unfamiliar strangers, the so called dear enemy phenomenon. However, in other species individuals are more, not less aggressive towards their neighbours. It has been hypothesised that this is due to the fact that neighbours can impose a greater threat than strangers, but this has not been tested previously.

**Results:**

We tested aggression in wild group-living male striped mice in a neutral test arena and demonstrate that breeders are more aggressive than non-breeding philopatrics, and that more aggression occurs during the breeding than during the non-breeding season. Male breeders were significantly more aggressive towards their neighbours than towards strangers, leading to the prediction that neighbours are the most important competitors for paternity. Using a molecular parentage analysis we show that 28% of offspring are sired by neighbouring males and only 7% by strangers.

**Conclusions:**

We conclude that in male striped mice the main function of male aggression is defending paternity against their territorial neighbours.

## Background

Territoriality functions to monopolize resources including food, shelter and access to mates, and is thus a strategy to increase fitness [1,2]. The importance of territoriality in obtaining reproductive success has been demonstrated for example in coyotes (*Canis latrans*) where reproductive success within a population was obtained exclusively by territorial individuals [3]. While territoriality can have significant benefits, it is also costly [4], especially in forms of energy expenditure [5], time requirements and the increased risk of injury [6] and predation [1].

As territoriality is costly, it is not surprising that individuals seek strategies to reduce these costs. One possibility is to reduce territorial aggression towards individuals that are less likely to pose a threat. For example in many species males show less or no aggression towards strange females compared to males [7-13], as females represent potential mates rather than competitors [14]. Another example is the "dear enemy" phenomenon, a case of context-specific territorial response, where territory holders are less aggressive towards familiar neighbours than towards strangers [15,16]. It is believed that floating (= unfamiliar) males pose a greater threat as they seek to obtain a territory, while neighbouring territorial males accept each other's territory boundaries [16].

In contrast to the dear enemy phenomenon is the nasty neighbour phenomenon [17]: in some species, individuals are more aggressive towards their neighbours than towards strangers. It has been suggested that this is the case when neighbours represent a greater threat to territory holders than to strangers [15], but what exactly represents that increased threat is seldom known [17,18]. In red-winged blackbirds (*Agelaius phoeniceus*), which show the dear enemy and not the nasty neighbour phenomenon, it has been shown that males are more aggressive towards sexually attractive neighbours [19]. However, up to date direct evidence that the nasty neighbour phenomenon is due to stronger competition for paternity by neighbours is missing.

In the current field study we measured male aggression in neutral test arenas in the striped mouse (*Rhabdomys pumilio*). The striped mouse is group living with one single breeding male and up to 4 communally breeding females per group [20]. Groups typically contain several philopatric adult sons (and daughters) that are believed not to breed in their natal group [21] and all group members participate in territorial defence [22]. We predicted that male aggression is related to defending paternity in harems, i.e. that breeding males would be more aggressive than natally philopatric males and that male aggression is more pronounced during the breeding than during the non-breeding season. Then we tested for the dear enemy phenomenon by staging encounters between specific individuals. We found that breeders were more aggressive towards their neighbours, which would be in agreement with the nasty neighbour hypothesis. We then predicted neighbours to be the most severe competitors for paternity and tested this using molecular markers to determine paternity of 119 pups born in 9 different social groups, demonstrating extra-group paternity to be high and mainly due to neighbours. Finally, we demonstrated that female choice plays a role in the loss of paternity by the breeding male.

## Materials and methods

### Study area and period

The study was conducted 2004 to 2007 in Goegap Nature Reserve in South Africa (S 29 41.56, E 18 1.60). The area is arid, with an average rainfall of 160 mm p.a., and the vegetation type is classified as Succulent Karoo. The study received clearance from the animal's ethics committee of the University of the Witwatersrand (2004/87/2A and 2005/82/4).

### Study species

Striped mice are diurnal, inhabit an open habitat and are readily habituated to the presence of observers, which allows direct behavioural observations in the field [23]. The breeding season of 3-4 months occurs in spring from August to November (2-3 litters per female, [24]. Males follow one of three tactics [21]: (i) group-living territorial breeding males. These are the breeding males of extended family groups with up to four communally breeding females and several adult philopatric males and females. Groups can contain up to 30 adult individuals of both sexes but only one breeding male [20]. (ii) Group-living philopatric males that stay in their natal group after reaching adulthood. They might seek copulations with non-related females from neighbouring groups (as has been described for other species; [25,26]. (iii) Solitary roamers that try to breed with females of communal groups which are defended by breeding males. Roamers have much larger home ranges than other males, and their home ranges overlap the home ranges of several females [21,27]. Roamers might first go through a phase of floating, when they leave their natal group and try to find a home range. In contrast to males, females are typically philopatric and do not disperse [20].

### Trapping, observation and radio-tracking

We studied between 9 and 20 focal groups from 2004 to 2007, with a similar number of non-focal neighbouring groups. Mice from focal groups were trapped, marked, observed and radio-tracked to determine social tactics of males, as described in detail elsewhere [20,21,23], while mice from neighbouring groups were only trapped and marked. The tail tip was taken as tissue sample from each individual for genetic analysis and stored in 90% ethanol.

### Measuring aggression

To measure aggressive behaviour, trapped males were tested in a neutral presentation arena (100 × 80 × 65 cm) made of white veneered chipboard. The bottom was lined with plastic foil on which a 2-3 cm layer of sand was provided. The sand was obtained from the dry riverbed going through the field site. To avoid influence from olfactory cues from previous experiments, e.g. faeces or urine, the sand in the arena was changed between experiments and the arena was cleaned with 90% alcohol (for a similar procedure see [28].

Mice were tested in pairs and for each experiment each male was used only once as stimulus animal. A partition (79 × 47 × 1.5 cm chipboard) in the middle of the arena separated the two males for a habituation phase of 5 min. Seven sunflower seeds were given to each male during the 5 min habituation phase to calm down captured wild mice. All mice ate all of their sunflower seeds. Then the partition was removed and the striped mice were observed for 15 min. If the males showed severe aggression (biting), the experiment was immediately terminated before the 15 minutes had elapsed. We recorded all aggressive behaviours (see [22]: chasing, fighting (standing on their hind legs and kicking each other with their forelegs), and biting. Biting was always preceded by either chasing or fighting. We then calculated the frequency of aggressive interactions initiated by the focal male per minute. The aggressive behaviour patterns observed were the same as observed during field experiments and in a similar intensity and frequency [22].

Altogether we tested 27 dyads of breeding males during the breeding season (September and October 2004 and October 2005) and 16 during the non-breeding season in March 2006 and March 2007. 14 breeding males were tested twice during the breeding season (October 2004 and 2005), once with a breeding male neighbouring them, and the other time with a breeding male not neighbouring their territory. Six males were first tested with their neighbour, the other eight males firsts with the stranger. The average time between experiments a focal male was tested either with the neighbour or the stranger was 6.1 ± 5.1 days. Additionally, we tested 12 dyads of philopatric males during the breeding season 2005 with philopatric males unknown to them (not neighbours).

### Paternity analysis

We isolated DNA from mouse tissue using magnetic particle purification (BioSprint 96 DNA Blood Kit, Qiagen). We used 9 polymorphic microsatellite loci from the house mouse genome (Chr13_1, Chr1_12, Chr1_21, Chr2_3, Chr7_64, D3Mit211, Chr11_81, Chr19_18, Chr5_38), primarily from [29], and amplified them for all individuals in two multiplexes using the Qiagen PCR-Multiplex-Kit with a final concentration of 0.1/0.2 μM primer for 35 cycles at an annealing temperature of 60°C). Mean number of alleles per locus was 13.9 (range 10 - 18). Typing error rates for the nine loci were estimated as 0.014 and were strongly influenced by poor repeatability of amplification of one locus in one 96-well plate; if this one plate were excluded from the average, the average error rate then fell below 0.01. The proportion of loci typed was 0.97.

Parentage analyses were performed using Cervus 3.0. Parameters for the simulation of parentage analysis were set as 100,000 offspring, 95% sampling of candidate mothers, 85% sampling of candidate fathers, 0.015 proportion of loci mistyped (to be conservative), and the confidence level was set at 95%. We accepted parentage assignment when trio confidence was 95% and there was zero or one mismatch between each parent and offspring, and no more than two mismatches in the trio of candidate parents and offspring. If trio confidence was less than 95% but a parent-offspring pair met the 95% confidence threshold with one or fewer mismatches, we accepted the maternity or paternity. If both a mother and father of the same offspring could be separately assigned with 95% confidence and one or fewer mismatches, but the trio had a confidence value of less than 95% and/or had more than two mismatches, we awarded parentage to the putative father if its pair delta value with the offspring exceeded that of the putative mother, and *vice versa*.

Altogether, we analysed 119 pups born between 1^st ^September and 1^st ^December 2005 from 9 different social groups. All breeding females and breeding males from these groups were considered as potential parents, as well as all roaming males and breeding males from neighbouring non focal groups (total of 72 males). Tenure of breeding and roaming males at the field site was 2.1 ± 1.0 months. No neighbouring male took over a breeding position at any group during the study period. The breeding male which was present 3 weeks before birth of pups was regarded as a potential father, as parturition is approximately 3 weeks [24]. Additionally, 37 philopatric males of focal groups born at the start of the breeding season were considered as potential fathers for offspring born later in the breeding season.

We excluded 4 offspring from all further analysis, because their mothers were statistically unknown (all candidate mothers had negative loglikelihood scores) and group association of pups could not be confirmed by observations. Of the remaining 115 offspring, success in paternity assignment was 80.8%. We further excluded 14 offspring from statistical analysis whose father was most likely the breeding male of the groups or a neighbouring male (positive LOD score), but did not meet the confidence threshold. Thus, these offspring could not be confirmed as having been sired by an unknown male.

### Data analysis

Data are presented as mean ± SE. Data from aggression tests were analysed using non-parametric statistics, because sample sizes were small. The Mann-Whitney U-Test (U) was used for unpaired data, the Wilcoxon matched-pairs signed-ranks test for paired data (T). We used Fisher's Exact test for comparisons of ratios. We estimated multiple paternity and paternity share using maximum likelihood and estimated their confidence intervals by bootstrapping 100,000 times, using the method of Eccard and Wolf [30] in R 2.9.1. Multiple paternity estimates the proportion of litters sired by more than one male. However, larger litters are more likely to show multiple paternities than smaller litters [30]. We therefore also used a second measure, paternity share, that is independent of litter size [30]. Paternity share is an estimate of the probability that an offspring is sired by a male other than the primary male. The median litter size of 3 was used as input, together with the empirically based estimates of multiple paternity in litters (see Results).

## Results

### Is male aggression related to reproduction?

During the breeding season, breeding males showed 2.0 ± 0.7 aggressive interactions/min, while philopatric males did not show any aggression (0.0 ± 0.0 aggressive interactions/min). During the breeding season, 16 of 27 trials had to be terminated due to high aggression, and 3 of 16 during the non-breeding season (p < 0.02, Fisher's Exact test). As the standard deviation for philopatric males was zero, we performed a Fisher's Exact test: 21 of 27 breeding males showed aggression but none of the 12 philopatric males (p = 0.0002). Breeding males showed significantly more aggressive interactions during the breeding (2.0 ± 0.7 aggressive interactions/min, N = 27) than during the non-breeding season (0.2 ± 0.1 aggressive interactions/min, N = 16; p = 0.03, U = 130.50).

### Dear or nasty neighbour?

**Figure 1:**
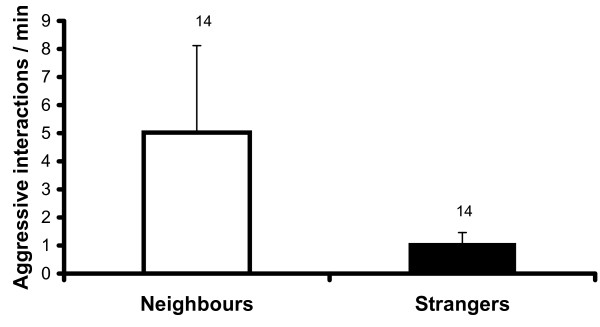
**Aggressive interactions initiated by breeding males in neutral arena tests during the breeding season towards neighbouring breeding males or towards breeding males not neighbouring them ("strangers")**. Mean, SE and sample sizes are shown. p = 0.001.

Breeding males tested during the breeding season showed significantly more aggression towards their neighbours (5.0 ± 3.1 aggressive interactions/min) than towards strange breeding males not neighbouring them (1.1 ± 0.4 aggressive interactions/min; p = 0.001, T = 0; paired N = 14; Fig. 1). Nine trials with neighbours and 6 trials with strangers had to be terminated (p > 0.4, Fisher's Exact test)., and the total duration of the experiments until termination (maximum 900 seconds) was shorter with neighbouring males than with strangers (412 ± 404 seconds *versus *611 ± 380 seconds; p < 0.02, T = 3).

### Multiple paternities and extra-group paternity

Of 24 litters, 15 (62.5%) had only one father, 8 had two fathers (33.3%) and one had three fathers (4.2%). Multiple paternity of litters was estimated as 36.0% (95% confidence interval: 16.0% - 56.0%). An alternative estimate of multiple paternity, the paternity share [30], was estimated as 13.9% (95% confidence interval: 5.6% - 24.8%).

**Figure 2:**
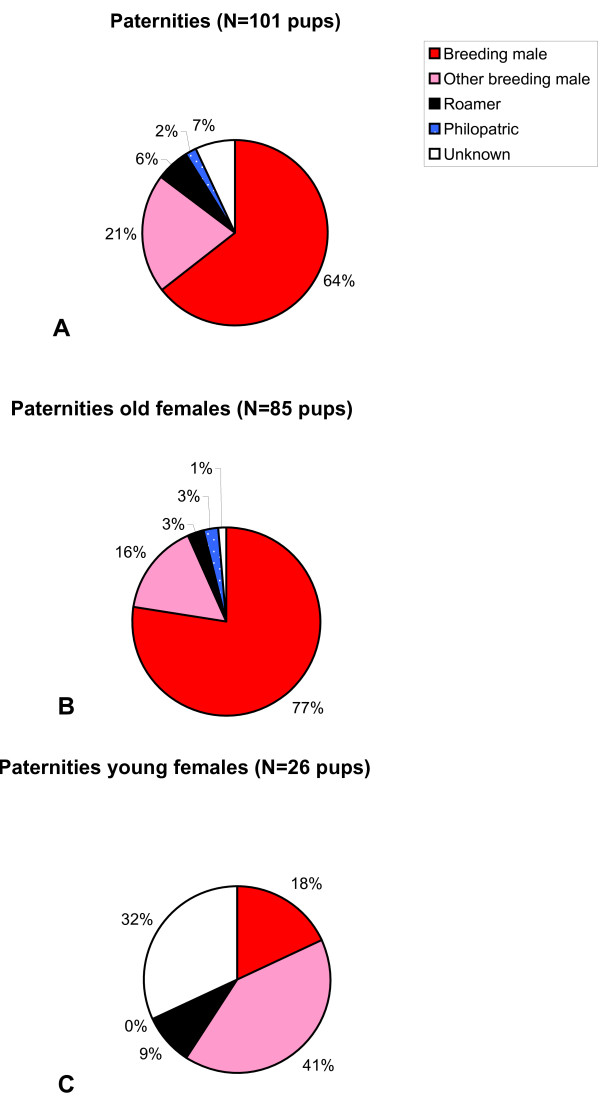
**Paternity within groups by the breeding male of the group, neighbouring breeding males, roamers, philopatrics and unknown males, for A) entire groups, B) old breeding females, C) young philopatric females**.

Of the 101 pups, 65 (64.4%) were sired by the breeding male of the group and 36 (35.6%) by other males. Neighbouring males sired altogether 28 pups (27.7%) (Fig. 2a). These males consisted of neighbouring breeding males (sired 21 pups or 20.8%), roamers (sired 6 pups or 5.9%) and one neighbouring philopatric male (sired 1 pup or 1.0%). Seven pups (6.9%) were sired by unknown males. Taking social group as the unit of analysis, significantly more extra-group young were sired by neighbouring males (3.0 ± 2.4 pups) than by unknown strange males (0.8 ± 1.4 pups; paired t_8 _= 2.53, p = 0.035). Only one pup was sired (1.0%) by a natal philopatric male (Fig. 2a).

### Comparison between young and old breeding females: indication for active female choice

In 6 of the 9 groups, some philopatric young females born at the start of the breeding season reproduced at the end of the breeding season. Altogether 14 of the 50 young females born between July and October 2005 bred, while 18 of the 20 old females born between September 2004 and June 2005 bred. Significantly more old than young females were reproductive (Fisher's Exact test, p < 0.0001). Young breeding females differed from the old breeding females in the pattern of extra-group paternities (Fig. 2b and 2c). The breeding males of the groups were the father of 79.6 ± 17.2% of the offspring of old females, but only of 12.5 ± 20.9% of the offspring of young females (paired t_5 _= 6.624, p < 0.01; Fig 2b and 2c). Taking social group as the unit of analysis, significantly more extra-group young were sired by neighbouring males (1.8 ± 1.6 pups) than by unknown strange males (0.1 ± 0.3 pups) for old breeding females (paired t_8 _= 3.162, p = 0.01), but not for young breeding females (1.2 ± 1.3 pups *versus *0.8 ± 1.4 pups; paired t_8 _= 1.51, p = 0.17).

Of the 14 young philopatric females that bred we could determine the father for 13 using the same microsatellites. For 12 of these females, the father was either still present as breeding male in their group (8 females), or their father was a neighbouring male that was still present (4 females). Only 2 of the 8 young females whose father was still the breeder of the group reproduced with him, while 18 of the 20 old breeding females reproduced with the breeding male of their group (Fisher's Exact Test, p < 0.002).

## Discussion

In the present study we showed that breeding male striped mice were more aggressive during the breeding season than during the non-breeding season. Further, the neighbours of breeding males appear to pose a recognisable threat to the breeding male's confidence of paternity and direct fitness. This threat explains the occurrence of the nasty neighbour phenomenon in striped mice and the aggressive responses elicited when a breeding male encounters his neighbour.

Striped mice are territorial [20] and both breeders and philopatrics participate in territorial defence [22]. Here we showed that male breeders are more aggressive than male philopatrics that typically do not breed within their group (for a similar result in coyotes see [3]), though they might seek copulations with females from neighbouring groups. Additionally, breeding males were more aggressive towards strangers during the breeding season than during the non-breeding season. Together with a previous study showing that males are less territorial towards females than towards males [22], these results indicate that male aggression occurs within the reproductive context. While year-round territoriality by all group members might act to protect resources such as food and shelter [22], the additional increase in aggression in male breeders is best explained as a mechanism to keep rival males from their territory and by this from their mating partners.

Male striped mice distinguish between males neighbouring them and strange males, indicating that they can differentiate between familiar and unfamiliar individuals or might even have the ability to recognize individuals (as described in other rodents: [31,32]. Our study provides growing evidence that the nasty neighbour phenomenon is more common than previously believed, as males were nearly five times more aggressive towards their neighbours than towards strangers. This indicates that male striped mice can alter their territorial behaviour which most likely depends on a pay-off asymmetry [33]: breeders have paternity to lose while philopatrics do not, and neighbours represent a stronger threat than strangers.

In territorial birds, neighbouring males often represent the greatest risk for loss of paternity to territory holders [34,35]. The same holds true for the striped mouse, where about 28% of pups are sired by neighbours. These were primarily neighbouring territorial males, but also solitary roaming males and to a much lesser extent philopatric males of neighbouring groups. On average, much more paternity was lost to territorial breeders than the other two categories of neighbouring males (Fig. 2). In another study we show that reproductive success of roamers is ten times smaller than that of territorial breeders, with philopatrics having even a hundred fold less reproductive success (Schradin and Lindholm, unpubl. data). In contrast, potential floating males that would be unfamiliar to striped mice played a minor role in extra-group paternity. In conclusion, neighbouring males and especially neighbouring territorial breeders induce high direct fitness costs for male breeders in the striped mouse, which use territorial aggression as a strategy to reduce these costs and defend paternity within their harems.

36% of litters had two or three fathers and the paternity share, an offspring-based measure of extra-group paternity independent of litter size [30] was 14%. High levels of multiple paternity are common in small mammals, both in polygynous [30] and socially monogamous species [36,37]. A paternity share of 14% is similar to the one observed in socially monogamous prairie voles (16%), but lower than the average of 21% reported for rodents and much lower than that of promiscuous rodent species (30-50%) [30]. Multiple mating by females is likely to represent active female choice [38] and can increase female fitness [39,40]. As females of one harem have synchronous oestrus but striped mice are solitary foragers [23], breeding males cannot continuously defend all their females, and the best strategy to defend paternity is to defend the territory and to keep neighbouring males away.

We found strong support for active female choice when comparing old breeding females born before the breeding season and their young adult philopatric daughters born at the start of the breeding season. Young females showed a much greater amount of extra-group paternity than old females (87% *versus *20%). This was mainly due to paternity obtained by neighbouring males as well as unknown males. These data can best be explained by inbreeding avoidance. It is known from a captive study that female striped mice do not breed with the adult male with which they grew up, independent of whether this is their biological or foster father [41]. This indicates that familiarity is the mechanism by which female striped mice avoid inbreeding which is in accordance with our results: presence or absence of the biological father had no effect on young females' mate choice. Most young females chose a male outside of their natal group for mating, even if their biological father was a neighbouring male. The average tenure of breeding males of more than 2 months is long enough that inbreeding between them and their daughters could occur, as females can start breeding when 4-6 weeks old females [27]. These results can also explain why extra-group paternity was much greater than the paternity share in our study, even though it has been argued that both should measure the same [30]. The two measurements are only analogues when the primary male of the paternity share is also the social male of the group, but not when the primary male is from outside the group, as is the case for young breeding females. This might be important for many other cooperatively breeding species where subdominant females mate with males from outside the group, for example in meerkats (*Suricata suricatta*; [25]). In sum, active female choice might be a prerequisite for the high success rate of neighbouring males in obtaining extra-group paternity.

Breeding males might not be able to defend paternity of young breeding females which, in contrast to old breeding females, seem to seek copulations with other males, maybe to avoid inbreeding. In fact, it could increase the breeding male's fitness if his daughters breed with other males. However, the pattern that neighbours pose a greater risk than strangers was not created by the mate choice behaviour of young females. It is for the old females that the risk of losing paternity to neighbours versus strangers is the highest. Therefore we conclude that neighbours pose the highest risk for breeding males, and that strangers can have significant success with young females.

## Conclusions

Müller & Manser [17] predicted that nasty neighbours are more common in social species that permanently stay in large groups with intensive competition between neighbouring groups. In contrast to banded mongooses (*Mungos mungo*), which also show the nasty neighbour phenomenon and forage in groups [17], striped mice are solitary foragers [23]. Thus, striped mouse males would always only meet a single other male at one time [23] independent of whether this male would be a neighbour or a stranger. Thus, it is the threat of the single individual, not the entire neighbouring group to which male striped mice respond. The dear enemy phenomenon has been described as a kind of cooperation between non-kin [19], which requires that benefits must be greater than costs (b>c) to occur. Thus, a high risk of reduced fitness by neighbours will increase benefits of territoriality. We predict that with increasing rate of extra-pair fertilizations by neighbours, animal species will rather show the nasty neighbour instead of the dear enemy phenomenon, a hypothesis that could be tested in songbirds, where data on extra-pair fertilizations are available from many species (e.g. [42]).

## Competing interests

The authors declare that they have no competing interests.

## Authors' contributions

C. Schradin designed the study, collected field samples and experiments and wrote the manuscript. C. Schneider collected data for the behavioural experiments. AKL did the genetic analyses and contributed to writing the manuscript. All authors have read and approved the final manuscript.
